# Distinguishing subclinical from clinical keratoconus by corneal measurements

**DOI:** 10.3389/fmed.2024.1427666

**Published:** 2024-11-14

**Authors:** Cristina Ariadna Nicula, Karin Ursula Horvath, Ariadna Patricia Nicula, Adriana Elena Bulboacă, Sorana D. Bolboacă, Dorin Nicula

**Affiliations:** ^1^Department of Oral-Maxilo-Facial Surgery, “Iuliu Hațieganu” University of Medicine and Pharmacy, Cluj-Napoca, Romania; ^2^Oculens Clinic, Cluj-Napoca, Romania; ^3^Department of Ophthalmology, Medicine and Pharmacy Science and Technology University, “George Emil Palade”, Târgu Mureș, Romania; ^4^Department of Ophthalmology, Emergency County Hospital, Târgu Mureș, Romania; ^5^Department of Pathophysiology, “Iuliu Haţieganu” University of Medicine and Pharmacy, Cluj-Napoca, Romania; ^6^Department of Medical Informatics and Biostatistics, “Iuliu Haţieganu” University of Medicine and Pharmacy, Cluj-Napoca, Romania

**Keywords:** keratoconus, subclinical keratoconus, topographic indices, tomographic indices, biomechanical parameters

## Abstract

**Purpose:**

The study aimed to determine the stability of topographic and tomographic indices measured with Pentacam and to evaluate the biomechanical parameters measured with Corvis ST in the diagnosis of subclinical keratoconus (sKCN) and clinical keratoconus (KCN).

**Methods:**

This is a single-center cohort study with a retrospective review of topographic and tomographic indices and biomechanical parameters on adult patients with subclinical keratoconus (sKCN), clinical keratoconus (KCN), and healthy subjects (control group). The area under the receiver operating curve (AUC) was used to identify the cutoff values for evaluated indices able to distinguish between subjects with sKCN and those with KCN.

**Results:**

Seventy-six patients (76 eyes) in the sKCN group, 74 patients (132 eyes) in the KCN group, and 70 patients (140 eyes) in the control group were analyzed. Evaluated participants had similar age, but in the sKCN group, men were predominant (*p* = 0.0070). Significantly higher values in the KCN group of Front Kmax, ISV, IVA, KI, IHD, BAD_D, and lower values of TL and PRC (with excellent accuracy AUC > 0.9) were observed in the differentiation of KCN by controls. Similarly, excellent accuracies were obtained by Front Kmax, ISV, IVA, KI, IHD, KISA, I-S, BAD_D, and RMS-total with higher values in the KCN group and PRC and ARTmax with lower values in patients with KCN as compared to those with sKCN. Only Front Kmean (AUC = 0.946, Se = 85.6%, Sp = 90.4%, *p* < 0.0001) and I-S Pentacam (AUC = 0.96, Se = 84.1%, Sp = 97.3%, *p* < 0.0001) proved accurate and not shared with differentiation of sKCN or KCN by normal eyes. Front Kmean Pentacam proved good for case findings (0.806 [0.742 to 0.871]) and screening (0.712 [0.645 to 0.778]). I-S Pentacam performed excellent for case findings (0.826 [0.764 to 0.888]) and good for screening (0.758 [0.700 to 0.817]).

**Conclusion:**

Subclinical and clinical KCN shared common Pentacam parameters with excellent or good accuracy in distinguishing subjects with and without pathology, but Front Kmean and I-S Pentacam proved excellent or good for case finding and screening and are not shared with differentiation of the sKCN or KCN by the normal eyes. Furthermore, differentiation of sKCN by normal eyes could be done with KISA (Pentacam) and CBI (Corvis) parameters, but only CBI is not shared with KCN.

## 1 Introduction

Keratoconus (KCN) is a bilateral progressive corneal non-inflammatory ectatic disease, characterized by a conical corneal shape, myopia, irregular astigmatism, corneal thinning, and decreased visual acuity in late stages (1). The disease is diagnosed during puberty and swiftly advances in the range from 10 to 20 years (2). Early detection of the disease and subsequent prevention of risks are crucial factors in its progression (3). During the early stages, the visual acuity may appear normal, but the slit-lamp examination and corneal topography and tomography can detect the subtle alterations in corneal regularity and thickness (4). The lack of classical keratometry and slit-lamp signs is characteristic of subclinical KCN (sKCN), but patients with clinical KCN (5–8) display typical topographic aspects.

One of the most used diagnostic methods is performing corneal topography and tomography with Pentacam HR (OCULUS Optikgeräte GmbH, Wetzlar, Germany). Pentacam employs a rotating Scheimpflug camera and a monochromatic slit-light source to capture 100 images from 1 to 360 in 2 s. Corneal tomography provides several quantitative indices with high specificity and sensitivity in the diagnosis of KCN (1, 9). Multiple studies indicate that specific Pentacam indices show promise in positively diagnosing subclinical and clinical KCN, yet there is no consensus on the appropriate cutoff values (9–12). Researchers have reported reproducible values for corneal thickness and posterior elevation (13, 14).

Corneal biomechanical parameters showed performances in the diagnosis of KCN (15). Corvis ST (OCULUS Optikgeräte GmbH, Wetzlar, Germany) is a device that combines the Pentacam parameters with the biomechanical data (16) and records corneal deformation responses after the application of a standardized air puff (17). Corvis ST has been reported to detect biomechanical abnormalities in early KCN stages or sKCN (18).

The investigation of Pentacam indices is the primary focus of the limited scientific literature on sKCN and KCN in Romania’s population. Our previous report highlighted the abilities of some Pentacam indices in distinguishing between sKCN and KCN patients, with KISA% (AUC = 0.991, Se = 95.8%, Sp = 98.1% cutoff = 92.322; Se indicates sensibility, and Sp indicates specificity) and PCR (AUC = 0.986, Se = 98.8%, Sp = 96.2%, cutoff = 5.7 mm) as excellent markers both for case finding and screening (19). Our study aimed to determine the stability of reported Pentacam topographic and tomographic indices and to investigate the abilities of Corvis ST biomechanical parameters in distinguishing patients with clinical keratoconus (KCN) from those with subclinical (sKCN).

## 2 Patients and methods

The research took place at Oculens Clinic in Cluj-Napoca, Romania, and received approval from the clinic’s Ethics Committee (no. 6/2022). The applied procedures were in concordance with the Declaration of Helsinki. The Ethics Committee waived the requirement for informed consent.

### 2.1 Study design and patients

We conducted an observational analytic cohort study with retrospective anonymous data collection. We evaluated subjects who received medical care in the healthcare facility from January 2018 to October 2022 for eligibility. Patients who had abnormal findings in topography and tomography maps but showed no signs of disease on clinical examination were included in the subclinical KCN group (sKCN). The KCN group included patients diagnosed with keratoconus, whereas the control group (C) included subjects without sKCN or KCN who were eligible for refractive surgery. We classified the severity of KCN disease using Belin’s ABCD classification (20). Patients with corneal scars, previous ocular surgery, dry eye syndrome, history of trauma, glaucoma, connective tissue diseases, or those who wear contact lenses in the last month prior to examination were excluded. Pregnant women were also excluded from the study.

All patients received a complete standard ocular examination and had the imaging performed with the Pentacam (OCULUS Optikgeräte GmbH, Wetzlar, Germany) and Corvis ST (Oculus Optikgeräte GmbH, Wetzlar, Germany). The patients were requested not to wear contact lenses for 1 month before the examinations. During the corneal tomography examination, all patients were asked to fixate on the central target and not to blink during the Scheimpflug camera rotation. The researchers assessed corneal biomechanical properties using the Corvis ST device (software version 1.5r1902) with two examinations at a 15-min interval. The same expert assistant performed all imagistic evaluations during one visit, following the same examination protocol, and using the same device for.

### 2.2 Data collection

**Figure 1:**
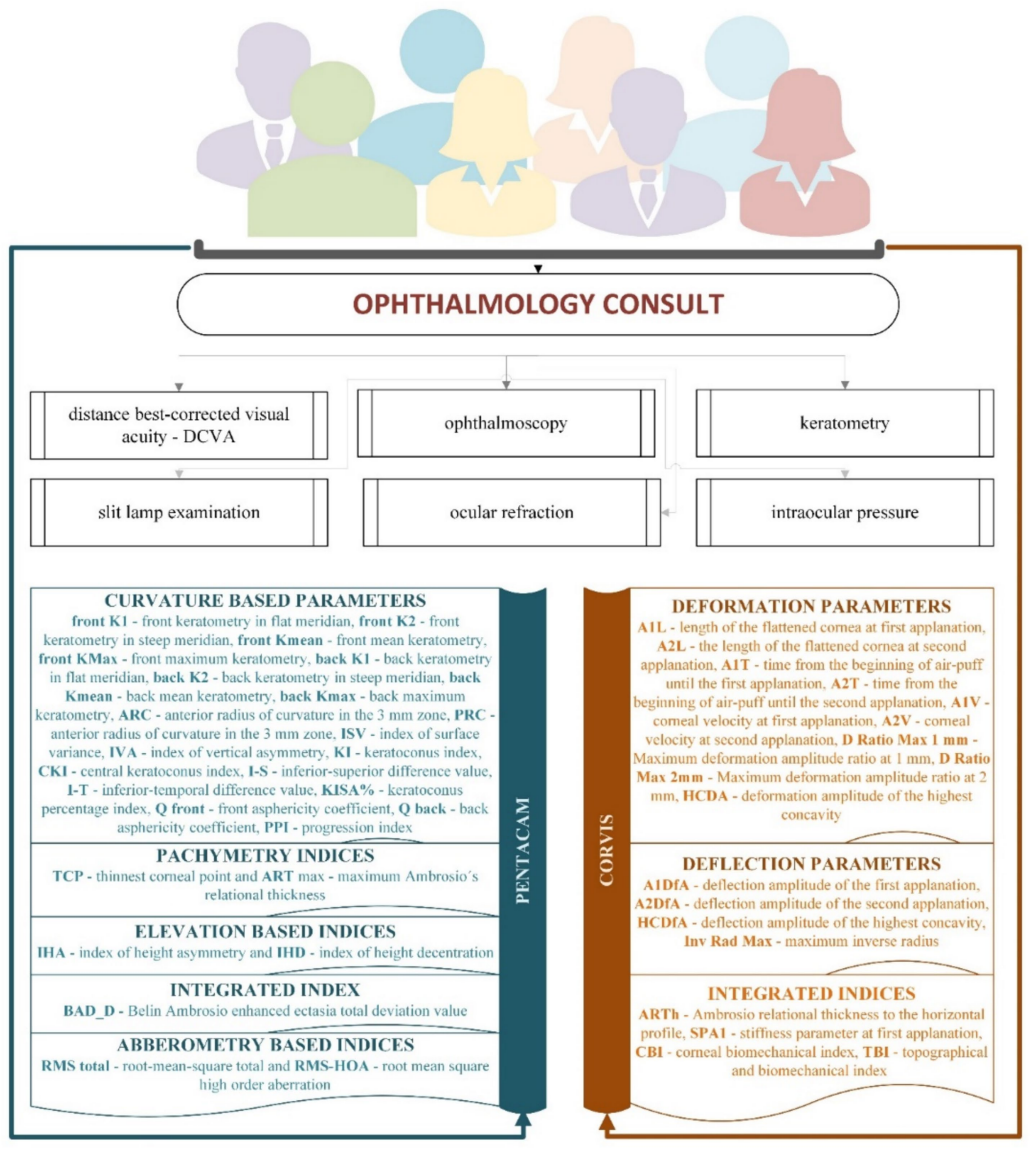
Clinical examination and data collection flow.

Figure 1 presents the flow of data collection. We evaluated 27 measurements retrieved from Pentacam (20 curvature-based indices, two pachymetry indices, two elevation-based indices, one integrated index, and two aberrometry-based indices) and 17 retrieved from Corvis (nine deformation, four deflection parameters, and four integrated indices).

### 2.3 Statistical analysis

The eye was the statistical unit in our analysis. The subject’s sex and number of evaluated eyes were summarized as absolute frequencies. Age, Pentacam, and Corvis parameters were first tested to identify the deviation from the theoretical normal distribution (Shapiro–Wilk test). Data were reported as median and [Q1 to Q3], where Q1 is the 25th percentile and Q3 is the 75th percentile, whenever the theoretical normal distribution proved violated. We used the Kruskal–Wallis test to compare the three groups and performed *post-hoc* analysis whenever we obtained statistical significance. The Mann–Whitney test was used to assess the differences between women and men. The indices measured with Pentacam and Corvis were the input data for receiver operating characteristic (ROC) curve analysis whenever significant differences between two groups (e.g., sKCN vs. KCN; sKCN/KCN vs. C) were observed. The performances of Pentacam and Corvis parameters in the classification of subjects were tested and AUC (area under the curve), Se (sensibility), Sp (specificity), for the cutoff values identified by the Youden index. We reported only AUC that indicated a good (>0.8) or an excellent (>0.9) model, considering the lower bound of a 95% confidence interval (21). We reported the clinical utility index for best-performing markers (http://www.clinicalutility.co.uk/, accessed 10 April 2024). Raincloud plots were used to illustrate the distribution of measurements between groups using the JASP program (v. 0.18.3.0). Raw data were analyzed with Statistics (v. 13.5, TIBCO Statistica, OK, USA) considering two-tailed tests and an adjusted significance level of 1.7%.

## 3 Results

Of 219 patients, 358 eyes were included in the analysis. Participants presented similar age (median values of 26 years for sKCN and 28 years for KCN and C; Kruskal–Wallis test: *p* = 0.6559) but statistically different distribution of sex (women: 19/76 (25%) in the sKCN group, 24/73 (32.9%) in the KCN group, and 41/70 (58.6%) in the C group; chi-squared test: *p* < 0.0001). Typically, women were older in the sKCN and KCN groups and younger in the C group (Table 1).

**Table 1:** Age and sex distribution by groups.

Group No. of subjects No. of eyes	Characteristics	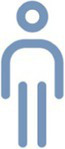	*p*-value	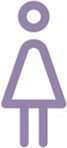
sKCN No. of subjects = 76 No. of eyes = 76	Sex^a^	57		19
	Eye^a^	57		19
	Age, years^b^	24 [19 to 32]	0.0070	31 [29 to 32]
KCN No. of subjects = 74 No. of eyes = 132	Sex^a^	49		24
	Eye^a^	86		46
	Age, years^b^	26 [21 to 33]	0.0743	30 [28 to 33]
Control No. of subjects = 70 No. of eyes = 140	Sex^a^	29		41
	Eye^a^	58		82
	Age, years^b^	31 [21 to 33]	0.4598	28 [21 to 33]

a Number.

b Median [Q1 to Q3], Q1—25^th^ percentile, Q3—75^th^ percentile; Mann–Whitney test.

As expected, the participants in our evaluated groups exhibit statistically significant differences in clinical characteristics (sphere (D), cyl (D), SE (D), and DCVA; *p*-values <0.0001, Table 2).

**Table 2:** Clinical characteristics by group.

Characteristic	sKCN	KCN	Control	*p*-value^*^
Sphere (D)	0.5 [0.3 to 1.3]	1.8 [0.8 to 3.3]	3.8 [2.8 to 4.8]	<0.0001
cyl (D)	0.8 [0.8 to 1.3]	2.6 [1.7 to 4]	1 [0.5 to 1.5]	<0.0001
SE (D)	1.3 [0.5 to 2.3]	3 [1.8 to 4.8]	4.4 [3.3 to 5.3]	<0.0001
DCVA	1 [0.8 to 1]	0.6 [0.4 to 0.8]	0.7 [0.7 to 1]	<0.0001

Data are expressed as median [Q1 to Q3] where Q is the quartiles. D, diopters; Cyl, cylinder; SE, spherical equivalent; DCVA, distance best-corrected visual acuity; sKCN, subclinical keratoconus; KCN, keratoconus; C, control group.

* Kruskal–Wallis test: post-hoc analysis with significant differences between groups (sKCN vs. KCN, sKCN vs. C, KCN vs. C), except for sKCN vs. C for Cyl.

### 3.1 Pentacam and Corvis characteristics by group

Curvature-based, elevation-based, pachymetry-based, integrated, and aberrometry-based indices measured with Pentacam showed statistically significant values between groups (Table 3).

**Table 3:** Variation of Pentacam between groups (KCN, sKCN, and C groups).

	sKCN group	KCN group	C group	*p*-value
Curvature-based indices				
Front K1 (D)	42.1 [41.5 to 43.3]	44.9 [43.2 to 47.3]	42.7 [41.9 to 43.8]	<0.0001
Front K2 (D)	44 [42.7 to 45.1]	47.8 [46.2 to 51.2]	44.4 [43.3 to 45.4]	<0.0001
Front Kmean (D)	43.1 [42.1 to 44.1]	46.2 [44.6 to 48.7]	43.5 [42.7 to 44.8]	<0.0001
Front Kmax (D)	44.8 [44.1 to 46.1]	53.5 [49.8 to 58]	44.7 [43.9 to 45.8]	<0.0001
Back K1 (D)	−6.1 [−6.2 to −6]	−6.4 [−6.9 to −6.1]	−6.2 [−6.3 to −6]	<0.0001
Back K2 (D)	−6.4 [−6.7 to −6.3]	−7.1 [−7.6 to −6.8]	−6.5 [−6.7 to −6.3]	<0.0001
Back Kmean (D)	−6.2 [−6.4 to −6.2]	−6.8 [−7.2 to −6.5]	−6.3 [−6.5 to −6.2]	<0.0001
Back Kmax (D)	−6.3 [−6.5 to −6.3]	−7.1 [−7.5 to −6.8]	−6.4 [−6.6 to −6.3]	<0.0001
ARC (mm)	7.8 [7.6 to 8]	6.8 [6.3 to 7.2]	7.7 [7.5 to 7.9]	<0.0001
PRC (mm)	6.2 [6 to 6.4]	5.2 [4.7 to 5.5]	6.3 [6.2 to 6.4]	<0.0001
ISV	24 [18 to 34]	76 [55.8 to 101]	17.5 [15 to 22.3]	<0.0001
IVA	0.22 [0.15 to 0.28]	0.87 [0.57 to 1.09]	0.11 [0.08 to 0.14]	<0.0001
KI	1.05 [1.02 to 1.07]	1.2 [1.13 to 1.3]	1.02 [1 to 1.03]	<0.0001
CKI	1.01 [1 to 1.01]	1.05 [1.02 to 1.07]	1.01 [1 to 1.01]	<0.0001
I-S value (D)	1.2 [0.6 to 2]	4.9 [3.7 to 7.6]	0.5 [0.1 to 0.7]	<0.0001
I-T	0.9 [0.8 to 1.1]	0.8 [0.6 to 1]	0.6 [0.5 to 0.7]	<0.0001
KISA %	57 [25.5 to 70.7]	351.8 [182.6 to 1,148]	3.6 [1.3 to 5.2]	<0.0001
Q front	−0.42 [−0.47 to −0.29]	−0.71 [−0.9 to −0.53]	−0.43 [−0.49 to −0.31]	<0.0001
Q back	−0.37 [−0.48 to −0.26]	−0.76 [−1.09 to −0.53]	−0.42 [−0.52 to −0.34]	<0.0001
PPI	2.1 [1.6 to 3.2]	1.9 [1.6 to 2.3]	0.06 [0.03 to 0.12]	<0.0001
Elevation-based indices				
IHA	5.2 [3.3 to 14.2]	31.8 [14 to 45.4]	4.4 [1.9 to 7.3]	<0.0001
IHD	0.02 [0.01 to 0.03]	0.11 [0.07 to 0.16]	0.009 [0.006 to 0.012]	<0.0001
Pachymetry-based indices				
TCP (μm)	524.5 [493.5 to 554]	467.5 [443.8 to 488.3]	547 [528 to 580]	<0.0001
ART-Max	336 [244 to 397]	172.5 [137.8 to 203.8]	443 [436 to 467]	<0.0001
Integrated index				
BAD_D	2.3 [1.4 to 3]	7.4 [5.7 to 10.3]	1.04 [1.02 to 1.06]	<0.0001
Aberrometry-based indices				
RMS-total	0.5 [0.4 to 0.7]	190.4 [181.9 to 206.5]	460 [402 to 514]	<0.0001
RMS-HOA	7.9 [7.8 to 8.1]	7.6 [5.8 to 10.5]	0.8 [0.4 to 1.3]	<0.0001

Data are expressed as median [Q1 to Q3] where Q is the quartiles. Front K1, front keratometry in flat meridian; Front K2, front keratometry in steep meridian; Front Kmean, front mean keratometry; Front Kmax, front maximum keratometry; back K1, back keratometry in flat meridian; back K2, back keratometry in steep meridian; back Kmean, back mean keratometry; back Kmax, back maximum keratometry; sKCN, subclinical keratoconus; KCN, keratoconus; ARC, anterior radius of curvature in the 3-mm zone; PRC, posterior radius of curvature in the 3-mm zone; ISV, index of surface variance; IVA, index of vertical asymmetry; KI, keratoconus index; CKI, central keratoconus index; I-S value, inferior–superior difference value; I-T, inferior–temporal difference value; KISA %, keratoconus percentage index; Q front, front corneal asphericity; Q back, back corneal asphericity; PPI, progression index; IHA, index of height asymmetry; IHD, index of height decentration; TCP, thinnest corneal point; ART-Max, maximum Ambrosio’s relational thickness; BAD_D, Belin–Ambrosio enhanced ectasia total deviation value; RMS-total, root mean square—total; RMS-HOA, root mean square—high-order aberration. Kruskal–Wallis test showed statistically significant differences in post-hoc analysis for (a) sKCN vs. KCN and KCN vs. control group (*p* < 0.0001) regarding Front K1, Front K2, Front Kmean, Front Kmax, Back K1, Back K2, Back Kmean, Back Kmax; (b) sKCN vs. KCN and KCN vs. C and sKCN vs. C for ISV, IVA, and KI (*p* < 0.0001); (c) for I-S, I-T, and KISA% values, for sKCN vs. KCN (*p* < 0.008, excepting PPI) and KCN vs. control group (*p* < 0.0001) and sKCN vs. control group (*p* < 0.0001 excepting Q front and Q back). Nine Corvis parameters showed significant differences between groups (Table 4).

### 3.2 Receiver operating characteristic curve analysis

**Figure 2:**
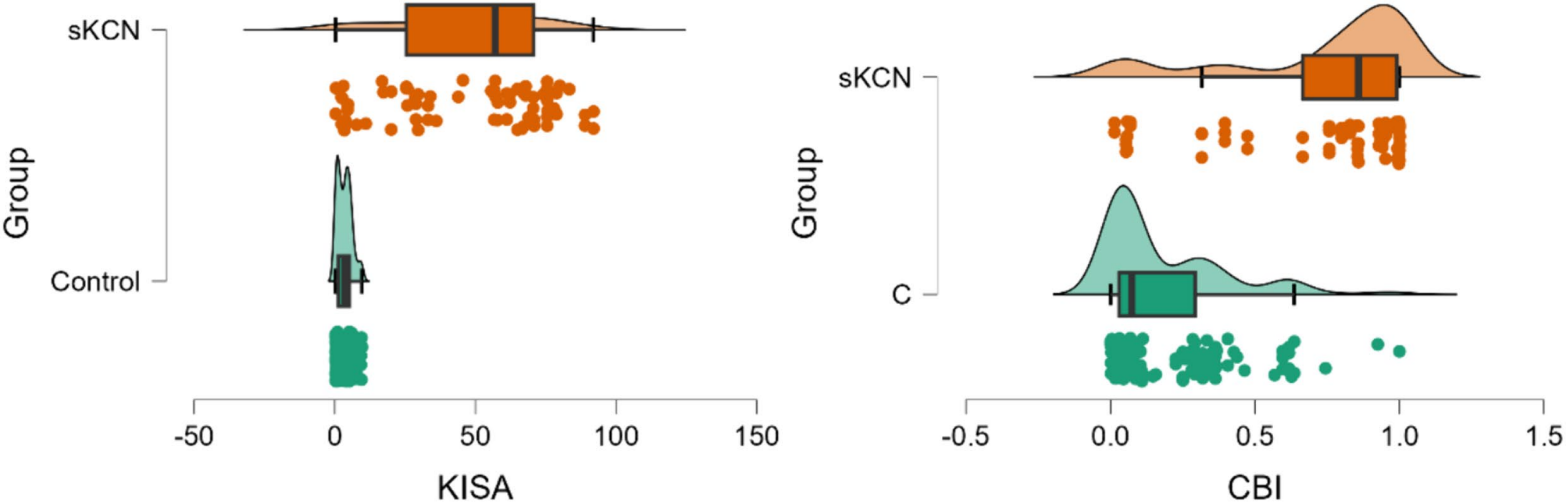
Distribution of KISA% and CBI values on patients with sKCN compared with controls. The dots represent the raw data, the box is determined by the value of the 25^th^ and 75^th^ percentile, and the line in the middle corresponds to the value of the median. The minimum and maximum values give the whiskers. Clinical performances in case finding (+CUI) or screening (−CUI): KISA%—excellent in case findings (0.816 [0.732 to 0.899]) and screening (0.909 [0.880 to 0.938]); CBI—good in case findings (0.726 [0.624 to 0.827]) and excellent in screening (0.865 [0.830 to 0.900]).

**Figure 3:**
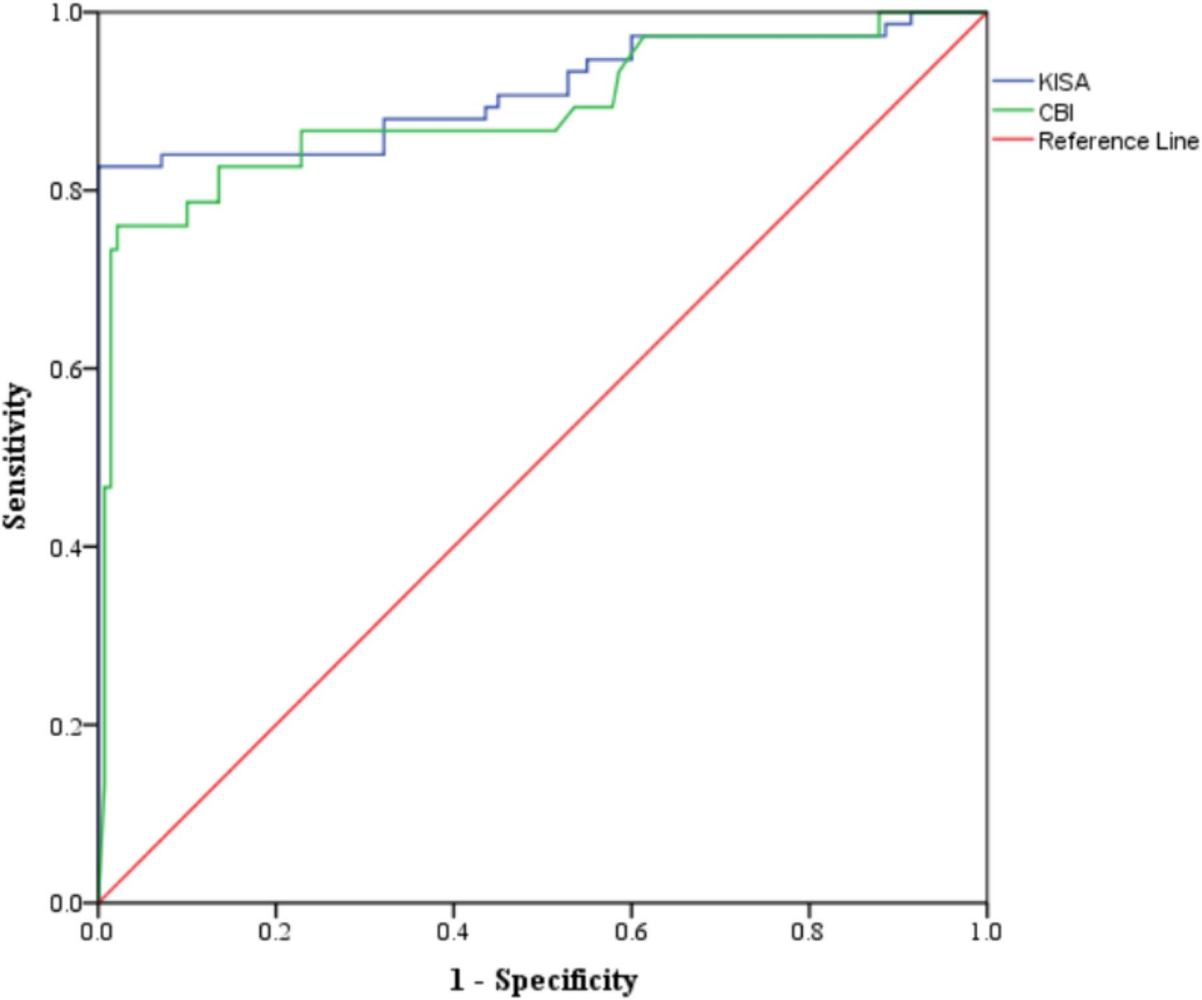
ROC analysis for markers with performances in the differentiation of sKCN by controls.

Four Pentacam and two Corvis parameters proved performances in the differentiation of sKCN by controls (Table 5; Figures 2, 3), but only the CBI is not shared with KCN. Three Pentacam (RMS-HOA, IP, and MS total) and one Corvis (TBI) parameter proved perfect classifies (AUC = 1; Table 5), showing an overfit and, therefore, the absence of performances on external raw data.

**Figure 4:**
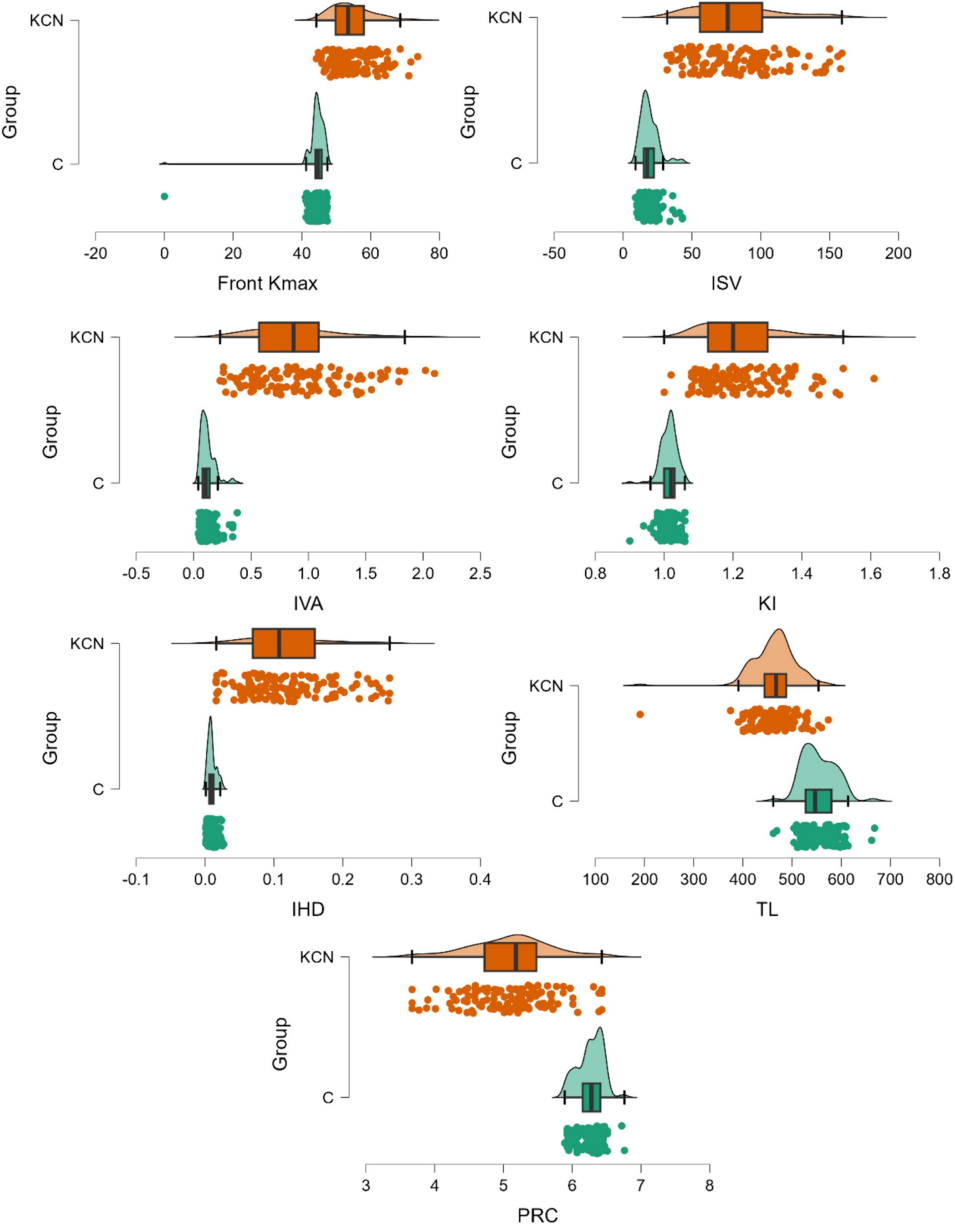
Distribution of markers on the KCN compared to the control group. The dots represent the raw data, the box is determined by the value of the 25^th^ and 75^th^ percentiles, and the line in the middle corresponds to the value of the median. The minimum and maximum values give the whiskers. Front Kmax—excellent in case findings (0.894 [0.846 to 0.942]) and screening (0.909 [0.880 to 0.938]); IVS and IVA—excellent in case findings (0.950 [0.917 to 0.982]) and in screening (0.950 [0.927 to 0.973]); KI—excellent in case findings (0.985 [0.967 to 1]) and screening (0.986 [0.974 to 0.998]); IHD—excellent in case findings (0.939 [0.903 to 0.976]) and screening (0.945 [0.922 to 0.968]); TL—excellent in case findings (0.851 [0.794 to 0.907]) and screening (0.867 [0.831 to 0.902]); PCR—excellent in case findings (0.917 [0.874 to 0.959]) and screening (0.927 [0.901 to 0.953]).

**Figure 5:**
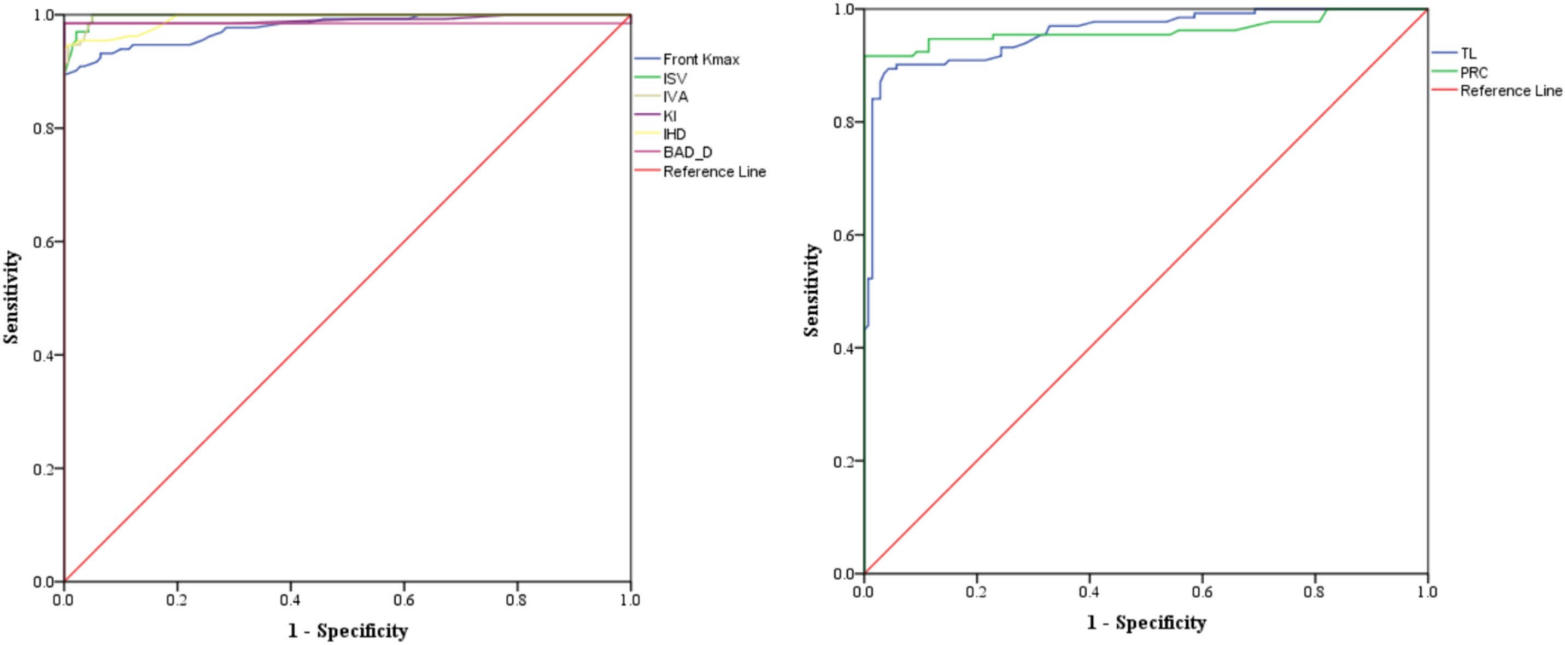
AUC for performing markers classified as excellent in the differentiation of KCN by controls. The graph on the left includes the markers with higher values in the KCN group, while the one on the right includes the markers with smaller values in the KCN group.

Eighteen Pentacam parameters (four overfit) and two Corvis parameters (two overfit) showed performance in the differentiation of KCN by controls, with some overlaps with sKCN (see Tables 5). The distribution of the markers in the KCN group compared to the control group for markers classified as excellent based on AUC is presented in Figure 4. Figure 5 presents the AUC for excellent markers in the differentiation of KCN by controls.

Eighteen parameters showed performances in discrimination between the KCN and sKCN groups (Table 6). Two markers, Front Kmean and I-S, are the only ones not shared with KCN vs. controls or sKCN vs. controls (Table 6).

**Figure 6:**
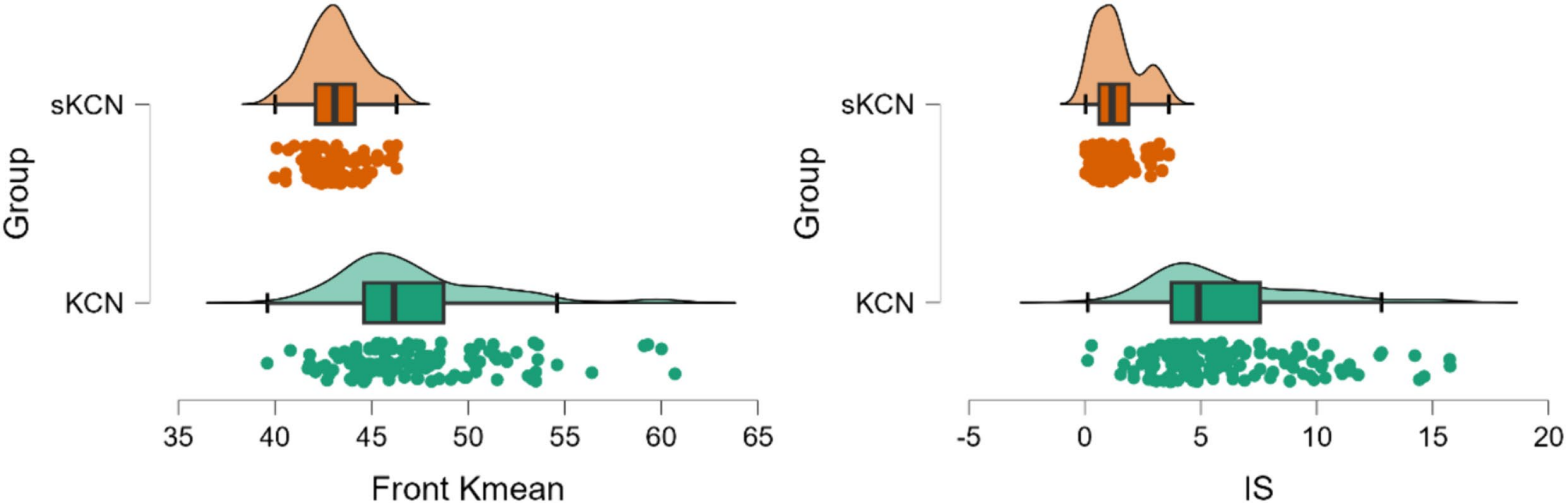
Distribution of performing measured markers in the KCN and sKCN groups. The dots represent the raw data, the box is determined by the value of the 25^th^ and 75^th^ percentiles, and the line in the middle corresponds to the value of the median. The minimum and maximum values give the whiskers. Clinical performances in case finding (+CUI) or screening (-CUI): Front Kmean—good for case findings (0.806 [0.742 to 0.871]) and screening (0.712 [0.645 to 0.778]) and I-S—excellent for case findings (0.826 [0.764 to 0.888]) and good for screening (0.758 [0.700 to 0.817]).

**Figure 7:**
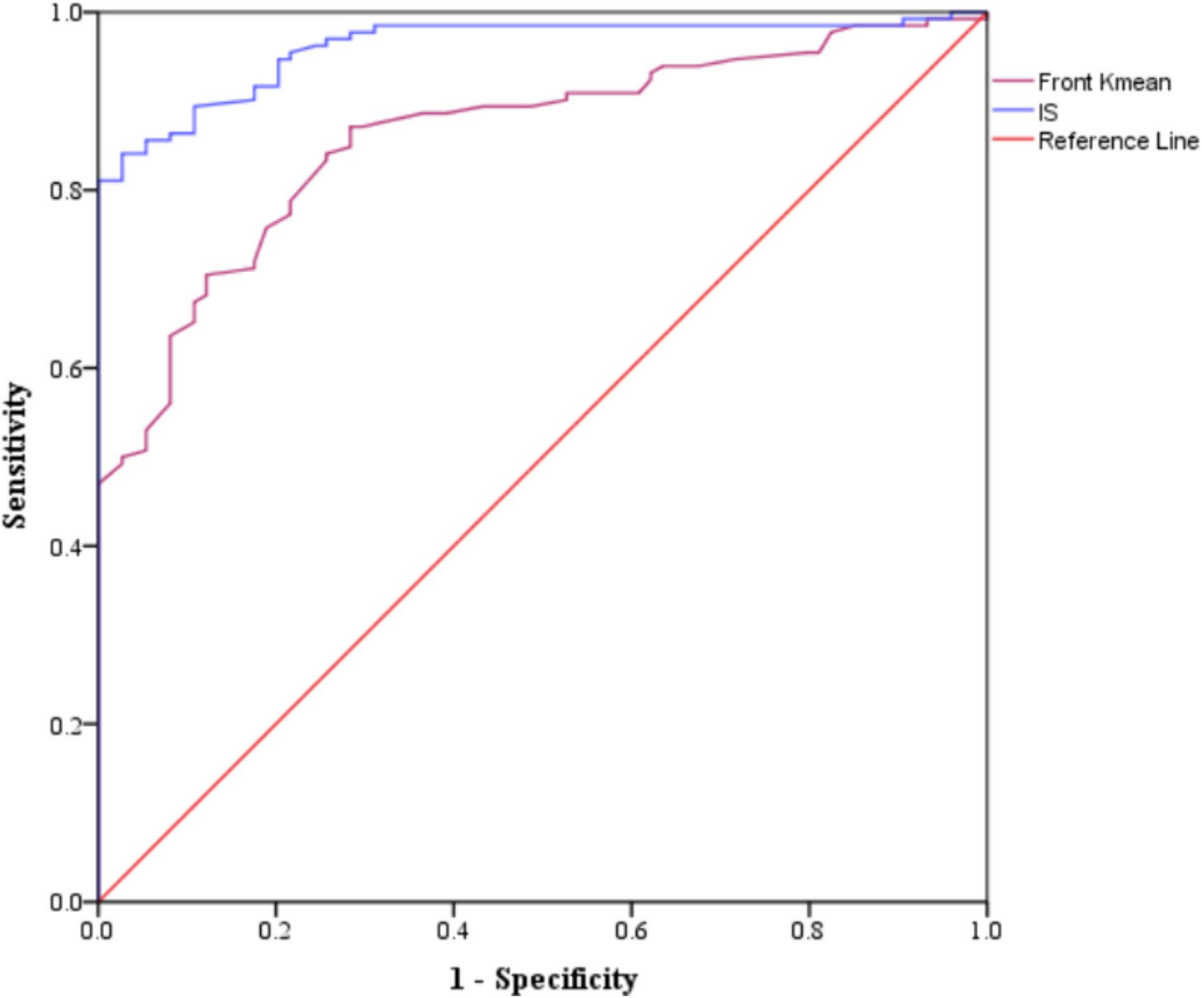
Areas under the curve for performing markers classified as good or excellent in the differentiation of KCN by sKCN.

The Front Kmean and I-S raw data exhibit higher values on patients with KCN than those with sKCN (Figure 6) and showed excellent performances in the differentiation of KCN by sKCN (Figure 7).

## 4 Discussion

In the evaluated cohort, Pentacam indices, and Corvis parameters demonstrated statistically significant differences between the investigated groups. Several Pentacam indices exhibit significant differences between pairs of groups, but most of the observed significant differences are shared by comparisons of pairs groups (Table 3) according to our *post-hoc* analysis. Only a limited number of indices exhibited excellent or good performance in distinguishing KCN or sKCN by controls or KCN by sKCN in ROC analysis (Tables 5, 6; Figures 2–7). The results of our study confirm that reported Pentacam indices are valid for diagnosing KCN and sKCN in a Romanian cohort, showing comparable performance in terms of AUC and cutoff values (19).

The anterior corneal curvature, pachymetry, and refractive status can predict the central posterior corneal curvature, excluding the corneal shape factor (22, 23). Keratoconus-suspect eyes showed discrepancies in corneal thickness and posterior corneal elevation values (24, 25). The biomechanical weakening of the cornea can help predict changes in the posterior corneal curvature (18). Consequently, the analysis of corneal biomechanics is critical for the early detection of KCN and other ectatic corneal diseases (26).

The Pentacam indices Front Kmax, ISV, IVA, KI, IHD, and BAD_D were identified in our study as excellent markers for differentiating KCN from normal corneas, with the KCN group exhibiting higher values and TL and PRC showing lower values (Table 5). According to Sedaghat et al. (27), I-S can distinguish between KCN and normal corneas. In contrast to our finding where KISA(%) proved overfit, Heidari et al. (15) reported KISA index is sufficiently strong for the differentiation of KCN compared to normal eyes (AUC > 0.8).

The results of our study indicate that IHA and IHD distinguished KCN from normal corneas, with good and excellent performances (Table 5; Figure 4). In previous reports, the IHD showed excellent performance in diagnosing KCN, with AUC values of 0.999 and 0.979 (15, 28). The study conducted by Tian et al. (16) revealed that IHD has a high discriminatory power (AUC = 0.999) and recommend to monitor patients with IHD higher than 0.008 and highlighted that IHD > 0.018 could indicate an increased risk of KCN. The Kmax, ISV, IVA, KI, IHA, and IHD previously demonstrated adequate strength to differentiate between KCN and sKCN eyes (AUC range: 0.83 to 0.981) (16). Kovács et al. (29) found that IHD was better at distinguishing KCN from normal corneas than BAD_D, which aligns with our findings of the study (Table 5). Hashemi et al. (30) found that IVA had good performances in diagnosis (AUC = 0.952), results also obtained in our study but with higher AUC and a different cut-off value (Table 5). The top three performing indices to differentiate KCN from normal corneas identified in our study are ISV, IVA, and BAD_D (Table 5; Figure 4). The utility of ARTmax has been reported as a valid diagnostic index for differentiating keratoconic eyes from normal corneas (31), without consistent value for determining the fruste form of KCN (32), while this index proved overfit in our study (Table 5). Tian et al. (16) demonstrated a low power for CBI Corvis index (AUC = 0.624) in differentiating KCN from normal corneas while other previously reported research evaluated the TBI (15, 33). In our study, only two Corvis indices proved significant differences in KCN and controls and were investigated, TBI that overfit and ARTh that exhibited only good performance (Table 5), so not necessarily recommended for clinical use.

Ren et al. (34) found that CBI had high diagnostic efficiency in differentiating KCN from sKCN and control eyes. The use of CBI is efficient in differentiating KCN from normal corneas (35) with clinical utility in screening. Sedaghat et al. (27) and Herber et al. (36) also found similar results, validating the clinical usefulness of CBI parameters to distinguish KCN from normal corneas. Francis et al. (37) found evidence suggesting that a parameter linked to corneal stiffness could be a valid measure for distinguishing KCN from normal eyes.

In our study, the CBI (Corvis) and KISA (Pentacam) showed good potential to diagnose sKCN when compared to normal eyes (Table 5). Our results are similar to those reported by Heidari et al. (15) with regard to KISA. Tian et al. (16) demonstrated a limited applicability of CBI to distinguish sKCN or KCN when corneas are ≤ 500 μm. Ambrósio et al. (38) found a sensitivity of 90% and specificity of 96% for a CBI (cutoff value of 0.29). Like Ambrósio et al. (38), Steinberg et al. reported CBI as a valid marker in the diagnosis of sKCN (35). Our findings showed that the Corvis TBI (Table 5) had an AUC equal with 1 showing on overfit and thus a parameter with possible external instability. Koc et al. (33) reported lower diagnostic accuracy for sKCN when the TBI Corvis index with a cutoff value equal to 0.29, with sensitivity at 67% and specificity at 86%.

The differentiation of KCN by sKCN was achieved in our cohort using 11 Pentacam parameters (Table 6; Figures 6, 7), with no Corvis parameters performing at a good or excellent level. Tian et al. (16) reported that ARTh and CBI provided moderate strength (AUC = 0.762, cutoff ≤ 338.03; AUC = 0.738, cutoff > 0.766) to distinguish KCN from sKCN.

In our cohort, the KCN group exhibited notable differences in A1T and D Ratio Max (1 mm) and D Ratio Max (2 mm) compared to the control group, as did the sKCN group (Table 4). Tian et al. (16) found A1T to be the predictor (AUC = 0.719) for sKCN, but the performance is limited. Our study also demonstrated significant differences (*p* < 0.0001) in all possible group comparisons for ARTh and SP-A1 (Table 4). Ren et al. (34) found that sKCN eyes have higher values for Max Inverse Radius (Inv Rad Max), D Ratio Max (2 mm), D Ratio Max (1 mm), Integrated Radius, and CBI than normal corneas, but lower than KCN eyes. The authors also suggested that SPA1 could be useful in distinguishing sKCN from normal corneas (34). Catalán-López et al. (39) found that combining A2L and corneal central thickness differentiated sKCN from normal corneas. In sKCN, the values of A1T, A1L SPA1, and HCR were reported to be lower than in the normal cornea (18, 40), while KCN exhibited higher values of A1V, A2T, A2V, and HCDA (16). Wu et al. demonstrated that HCR could differentiate between KCN, sKCN, and normal corneas (41). Our study revealed significant differences in A2DfA (mm) between sKCN and KCN, sKCN and control group, and a significant difference in Inv Rad Max for all group comparisons (Table 4). However, the observed differences do not show good or excellent discriminatory performances. Chan et al. (42) found that ART and Inv Rad Max had acceptable abilities to differentiate sKCN from control eyes, with AUC values of 0.836 and 0.754 (21), showing limited classification performance. Heber et al. (36) showed using regression analysis that the thinnest corneal thickness was accompanied by Max Inverse Radius, D Ratio Max (2 mm), D Ratio Max (1 mm), Integrated Radius, and SPA1 in normal and KCN eyes.

**Table 4:** Deformation parameters from Corvis and differences between groups.

Parameter	sKCN	KCN	C	*p*-value
Deformation parameters				
A1L (mm)	0.14 [0.13 to 0.15]	0.14 [0.13 to 0.14]	0.14 [0.13 to 0.15]	0.6118
A2L (mm)	0.36 [0.32 to 0.41]	0.35 [0.33 to 0.39]	0.36 [0.31 to 0.39]	0.3317
A1T (ms)	7.3 [7.1 to 7.6]	7.2 [6.9 to 7.8]	7.5 [7.3 to 7.8]	0.0001^*^
A2T (ms)	21.6 [21.5 to 21.8]	21.7 [21.3 to 22]	21.6 [21.4 to 21.9]	0.6406
A1V (mm/ms)	0.13 [0.11 to 0.14]	0.13 [0.11 to 0.17]	0.13 [0.12 to 0.14]	0.3415
A2V (mm/ms)	−0.25 [−0.28 to −0.23]	−0.26 [−0.3 to −0.24]	−0.26 [−0.28 to −0.24]	0.0374^#^
D Ratio Max (1 mm)	1.58 [1.55 to 1.7]	1.61 [1.56 to 1.7]	1.54 [1.5 to 1.58]	<0.0001^+^
D Ratio Max (2 mm)	4.17 [3.99 to 5.39]	4.45 [4.08 to 5.4]	4.01 [3.67 to 4.28]	<0.0001^+^
HCDA (mm)	0.97 [0.91 to 0.99]	0.99 [0.91 to 1.19]	0.96 [0.91 to 1.04]	0.0259
Deflection parameters				
A1DfA (mm)	0.1 [0.09 to 0.11]	0.1 [0.09 to 0.11]	0.1 [0.09 to 0.1]	0.1153
A2DfA (mm)	0.12 [0.11 to 0.13]	0.11 [0.1 to 0.12]	0.11 [0.11 to 0.12]	0.0083^**^
HCDfA (mm)	0.86 [0.77 to 0.87]	0.86 [0.77 to 1.05]	0.84 [0.77 to 0.91]	0.0837
Inv Rad Max (mm)	0.17 [0.16 to 0.2]	0.19 [0.18 to 0.22]	0.16 [0.14 to 0.17]	<0.0001^++^
Integrated parameters				
ARTh	270.6 [182.9 to 442.4]	279.7 [194 to 441.7]	525 [472.9 to 582.4]	<0.0001^++^
SPA1 (mmHg/mm)	100 [77.7 to 116.6]	93.5 [69.7 to 117]	116.2 [109.2 to 128.6]	<0.0001^++^
CBI	0.86 [0.67 to 0.99]	0.94 [0.15 to 0.99]	0.07 [0.03 to 0.29]	<0.0001^++^
TBI	1 [1 to 1]	1 [1 to 1]	0.22 [0.12 to 0.23]	<0.0001^++^

Data are expressed as median [Q1 to Q3] where Q is the quartiles. A1L(mm), the length of the flattened cornea at first applanation; A2L(mm), the length of the flattened cornea at second applanation; A1T, time from the beginning of air puff until the first applanation; A2T, time from the beginning of air puff until the second applanation; A1V, corneal velocity at first applanation; A2V (mm/ms), corneal velocity at second applanation; D Ratio Max 1 mm, maximum deformation amplitude ratio at 1 mm; D Ratio Max 2 mm, maximum deformation amplitude ratio at 2 mm; HCDA, deformation amplitude of the highest concavity; A1DfA, deflection amplitude of the first applanation; A2DfA, deflection amplitude of the second applanation; HCDfA, deflection amplitude of the highest concavity; Inv Rad Max, maximum inverse radius; ARTh, Ambrosio relational thickness to the horizontal profile; SPA1, stiffness parameter at first applanation; CBI, corneal biomechanical index; TBI, topographical and biomechanical index. Post-hoc analysis: *p < 0.005 for sKCN vs. C and KCN vs. C; ^#^*p* = 0.0411 for sKCN vs. KCN; ^+^*p* < 0.0001 for sKCN vs. C and KCN vs. C; **p < 0.04 for sKCN vs. KCN and sKCN vs. KCN; ^++^*p* < 0.000 for all possible comparisons between groups.

**Table 5:** Performant Pentacam and Corvis parameters in the differentiation of sKCN by controls and KCN by controls.

Parameter	Model	Cutoff	AUC [95%CI]	*p*-value	Se (%)	Sp (%)
sKCN vs. control—Pentacam						
KISA^+^	Good	10.350	0.913 [0.864 to 0.962]	<0.0001	82.7	100
RMS-HOA^+^	Overfit	4.485	1 [1 to 1]	<0.0001	100	100
IP^+^	Overfit	0.681	1 [1 to 1]	<0.0001	100	100
RMS-total^++^	Overfit	160.835	1 [1 to 1]	<0.0001	100	100
sKCN vs. control—Corvis						
CBI^+^	Good	0.651	0.891 [0.838 to 0.943]	<0.0001	76.0	97.9
TBI^+^	Overfit	0.708	1 [1 to 1]	<0.0001	100	100
KCN vs. control—Pentacam						
Front K2^+^	Good	46.050	0.881 [0.837 to 0.924]	<0.0001	77.3	90.0
Front Kmax^+^	Excellent	47.700	0.978 [0.963 to 0.994]	<0.0001	89.4	100.0
KISA^+^	Overfit	9.589	1 [1 to 1]	<0.0001	100.0	99.3
ISV^+^	Excellent	30.500	0.998 [0.995 to 1]	<0.0001	100.0	95.0
IVA^+^	Excellent	0.220	0.998 [0.995 to 1]	<0.0001	100.0	95.0
KI^+^	Excellent	1.065	0.991 [0.979 to 1]	<0.0001	98.5	100.0
IHA^+^	Good	10.300	0.898 [0.857 to 0.939]	<0.0001	82.6	90.0
IHD^+^	Excellent	0.026	0.993 [0.986 to 0.999]	<0.0001	94.7	99.3
BAD_D^+^	Excellent	1.395	0.985 [0.964 to 1]	<0.0001	98.5	100.0
IP^+^	Overfit	1.395	1 [1 to 1]	<0.0001	98.5	100.0
RMS-HOA^+^	Overfit	1.966	1 [1 to 1]	<0.0001	100.0	99.3
Back K2^++^	Good	−6.850	0.857 [0.809 to 0.906]	<0.0001	68.2	96.4
Back Kmax^++^	Good	−6.750	0.919 [0.882 to 0.956]	<0.0001	78.8	97.1
TL ^++^	Excellent	510.500	0.958 [0.935 to 0.981]	<0.0001	89.4	95.7
ARC^++^	Good	7.355	0.92 [0.879 to 0.96]	<0.0001	85.6	99.3
PRC^++^	Excellent	5.880	0.962 [0.935 to 0.989]	<0.0001	91.7	100.0
ARTmax^++^	Overfit	374.500	1 [1 to 1]	<0.0001	100.0	100.0
Q front^++^	Good	−0.515	0.858 [0.811 to 0.905]	<0.0001	78.0	85.7
KCN vs. control—Corvis						
TBI^+^	Overfit	0.708	1 [1 to 1]	<0.0001	100.0	100.0
ARTh^++^	Good	462.782	0.855 [0.809 to 0.901]	<0.0001	81.8	78.6

sKCN vs. control ^+^ large value in the sKCN group; ^++^ small value in the KCN group; KCN vs. control ^+^ large value in the KCN group; ^++^ small value in the KCN group; AUC, area under curve; SE, standard error; CI, confidence interval; Se, sensitivity; Sp, specificity; KISA, keratoconus percentage index; RMS-HOA, root mean square—high-order aberration; IP, index progression; RMS-total, root mean square—total; CBI, corneal biomechanical index; TBI, topographical and biomechanical index; Front K2, front keratometry in steep meridian; Front Kmax, front maximum keratometry; KISA, keratoconus percentage index; ISV, index of surface variance; IVA, index of vertical asymmetry; KI, keratoconus index; IHA, index of height asymmetry; IHD, index of height decentration; BAD_D=Belin/Ambrosio enhanced ectasia total deviation value; IP, index progression; RMS-HOA, root mean square—high-order aberration; Back K2, posterior keratometry on the steepest meridian; Back Kmax, posterior maximum keratometry; TL, thinnest location; ARC, anterior radius curve in the 3-mm zone; PRC, posterior radius curve in the 3-mm zone; ART max, Ambrosio relational thickness maximum; Q front, asphericity coefficient of the front surface; TBI, topographical and biomechanical index; ARTh, Ambrosio relational thickness to the horizontal profile.

**Table 6:** Performant Pentacam and Corvis parameters in the differentiation of KCN by sKCN.

Parameter	Model	Cutoff	AUC [95%CI]	*p*-value	Se (%)	Sp (%)
KCN vs. sKCN—Pentacam						
Front K2^+^	Good	45.750	0.894 [0.851 to 0.937]	<0.0001	79.5	89.0
Front Kmean^+^	Good	43.750	0.860 [0.810 to 0.910]	<0.0001	87.1	74.0
Front Kmax^+^	Excellent	48.650	0.946 [0.915 to 0.977]	<0.0001	85.6	90.4
ISV^+^	Excellent	40.000	0.966 [0.94 to 0.992]	<0.0001	95.5	87.7
IVA^+^	Excellent	0.345	0.95 [0.92 to 0.98]	<0.0001	94.7	83.6
KI^+^	Excellent	1.075	0.96 [0.934 to 0.986]	<0.0001	97.7	83.6
CKI^+^	Worthless	1.025	0.84 [0.787 to 0.894]	<0.0001	66.7	100.0
IHA^+^	Worthless	23.950	0.83 [0.776 to 0.884]	<0.0001	59.8	97.3
IHD^+^	Excellent	0.047	0.949 [0.923 to 0.976]	<0.0001	90.9	86.3
KISA^+^	Excellent	92.322	0.985 [0.969 to 1]	<0.0001	96.2	100.0
I-S^+^	Excellent	3.355	0.96 [0.935 to 0.985]	<0.0001	84.1	97.3
BAD_D^+^	Excellent	4.540	0.953 [0.919 to 0.988]	<0.0001	93.2	95.9
RMS-total^+^	Overfit	10.837	1 [1 to 1]	<0.0001	100.0	100.0
Back K2^++^	Good	−6.950	0.852 [0.802 to 0.902]	<0.0001	62.9	97.4
Back Kmax^++^	Good	−6.450	0.927 [0.891 to 0.962]	<0.0001	77.3	80.3
ARC^++^	Good	7.205	0.896 [0.845 to 0.947]	<0.0001	77.3	97.4
PRC^++^	Excellent	5.705	0.95 [0.922 to 0.978]	<0.0001	87.9	94.7
ARTmax^++^	Excellent	234.000	0.942 [0.914 to 0.97]	<0.0001	87.1	88.2

KCN by sKCN ^+^ large value in the KCN group; ^++^ small value in the KCN group; AUC, area under curve; SE, standard error; CI, confidence interval; Se, sensitivity; Sp, specificity; Front K2, front keratometry in steep meridian; Front Kmean, mean keratometry on the front surface; Front Kmax, front maximum keratometry; ISV, index of surface variance; IVA, index of vertical asymmetry; KI, keratoconus index; CKI=central keratoconus index; IHA, index of height asymmetry; IHD, index of height decentration; KISA, keratoconus percentage index; I-S, difference between the inferior and superior cornea; BAD_D=Belin/Ambrosio enhanced ectasia total deviation value; RMS-total, root mean square—total; RMS-HOA, root mean square—high-order aberration; Back k2, posterior keratometry on the steepest meridian; Back Kmax, posterior maximum keratometry; ARC, anterior radius curve in the 3-mm zone; PRC, posterior radius curve in the 3-mm zone; ARTmax, Ambrosio relational thickness maximum.

The strengths of our study are represented by the applied rigorous methodology and the existence of a control group, to compare the measured indices and to identify those markers with diagnostic potential. The evaluated number of patients was higher than our previous study (21), and the slight changes in the cutoff values for Pentacam indices indicate the robustness of our findings. Our findings show the potential of Pentacam and Corvis indices in differentiating patients with sKCN or KCN by controls, respectively, KCN by sKCN. However, their potential to become current practice need validation on external cohorts. While our study has strengths, we cannot overlook its limitations. The small number of participants in each group guarantees that our results directly reflect the evaluated cohort, but they must be interpreted with caution. To support generalizability, our results must undergo external validation. The study was done at one location, which may introduce institutional biases and limit the generalizability of the results. Multi-center studies would help to mitigate institutional biases and enhance the generalizability of the results. The retrospective nature of data collection hinders our ability to infer causality or track the development of sKCN or KCN over time. Long-term studies can effectively measure the changes in Pentacam and Corvis parameters during the disease progression. We concentrated on the Pentacam and Corvis parameters in our study, without considering additional diagnostic tests. Including a broader range of diagnostic tools (e.g., genetic, environmental, and behavioral) could provide a more comprehensive assessment. Furthermore, emerging technologies or a combination of different tools that increase the costs of diagnosis showed diagnostic performances (43–46), but validity and reliability assessment must provide evidence to support current practice implementation.

Lack of agreement is common in scientific literature regarding tomography and topography findings (43), potentially caused by variations in devices used (47, 48), genetic characteristics of the evaluated patients (49), and individual intrinsic biologic diversity. Consideration of all potential factors and confounders is crucial when determining cutoff values for diagnosing sKCN and KCN.

In conclusion, our findings showed that KISA (Pentacam) and CBI (Corvis) metrics are effective in differentiating sKCN from normal eyes in our evaluated cohort. To distinguish KCN from normal eyes, the most effective approach is to analyze ISV, IVA, IHD, KI, and BAD_D Pentacam measurements. Only two Pentacam indices, Front Kmean and I-S, have shown performances on the differentiation of KCN by sKCN not shared with distinguish between KCN and sKCN from normal eyes. Among the Corvis parameters, only ARTh showed performances in distinguishing KCN from normal eyes and CBI for distinguish sKCN from the normal eyes, but they did not reach the good or excellent thresholds for case findings or screening are not achieved.

## Data Availability

The data used to support the results of the present study are available from the corresponding author upon request.
